# An HWE‐Family Histidine Kinase Modulates *Brucella* Cell Envelope Properties and Host Innate Immune Response

**DOI:** 10.1111/mmi.70006

**Published:** 2025-06-26

**Authors:** Xingru Chen, Emily Perez, Eleanor C. Scheeres, Rosemary Northcote, Aretha Fiebig, Andrew J. Olive, Sean Crosson

**Affiliations:** ^1^ Department of Microbiology, Genetics, and Immunology Michigan State University East Lansing Michigan USA

**Keywords:** cell envelope, cytokine, histidine kinase, infection, intracellular pathogen, signal transduction, stress response

## Abstract

The bacterial cell envelope is essential for viability and host interaction. In the intracellular pathogen *Brucella ovis* , the orphan HWE‐family histidine kinase PhyK has been implicated in processes that influence cell envelope homeostasis, yet its function remains largely uncharacterized. We show that deletion of *phyK* (∆*phyK*) disrupts cell size control, increases resistance to anionic detergents, enhances sensitivity to cationic envelope disruptors, and triggers broad transcriptional changes, including reduced expression of aerobic respiration genes and increased expression of genes involved in transport and lipid metabolism. This transcriptional profile mirrors that of wild‐type *B. ovis* exposed to an anionic detergent, indicating that loss of PhyK function primes cells to resist this stress. Despite its altered cell envelope properties, the ∆*phyK* mutant exhibits no fitness defect in ex vivo macrophage infection models. However, it elicits a significantly reduced pro‐inflammatory cytokine response in activated murine macrophages compared to the wild‐type strain. We further show that purified PhyK can form multiple stable oligomeric species in solution, reflecting the structural plasticity observed in other HWE‐family kinases and likely contributing to its signaling function in vivo. Our results establish PhyK as a key regulator of *B. ovis* cell envelope properties that can modulate host immune interactions.

## Graphical abstract

The HWE‐family histidine kinase PhyK regulates the cell envelope structure of the intracellular pathogen *Brucella ovis* and affects how the host immune system responds to this bacterium. Deletion of *phyK* alters bacterial survival in the face of detergent stress and reduces immune activation in infected macrophages. This study identifies PhyK as an important regulator of *Brucella* cell envelope homeostasis and host interaction.

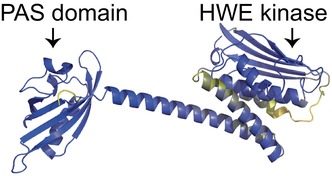

## 1 Introduction

*Brucella* spp. are Gram‐negative intracellular pathogens that infect a range of domesticated animals, wildlife, and humans (Godfroid et al. 2014; Laine et al. 2023; Pinn‐Woodcock et al. 2023). Among the six classical *Brucella* species, *B. ovis* is distinguished by its combination of an inability to synthesize O‐polysaccharide, a degraded genome containing an abundance of pseudogenes, and its lack of zoonotic potential (Rossetti et al. 2022; Tsolis et al. 2009; Wattam et al. 2009). *B. ovis* infection is associated with abortions in ewes and increased perinatal mortality (McFarlane et al. 1952; Osburn and Kennedy 1966), but its primary impact is on males. Specifically, rams can become persistently infected, resulting in epididymitis and diminished fertility (Biberstein et al. 1964; Carvalho et al. 2012). In the field, *B. ovis* infections are restricted to sheep, with farmed red deer being the only other animals confirmed to be naturally infected by this species (Ridler et al. 2000).

*Brucella ovis* was initially described as a “*Brucella* mutant” because of its non‐smooth (i.e., rough) phenotype on agar plates (Buddle and Boyes 1953), a characteristic resulting from the absence of O‐polysaccharide on its lipopolysaccharide (LPS). Enhanced exposure of outer membrane proteins (OMPs) due to the lack of O‐polysaccharide (Bowden et al. 1995) and the distinct OMP profile of the *B. ovis* cell envelope (Martin‐Martin et al. 2011; Tsolis et al. 2009) are hypothesized to influence *B. ovis* interactions with mammalian host cells in ways that set it apart from other classical *Brucella* species (Vizcaíno and Cloeckaert 2012). The unique envelope characteristics of *B. ovis* likely contribute to its heightened susceptibility to various membrane‐disrupting agents compared to other *Brucella* species (Freer et al. 1999; Martin‐Martin et al. 2011).

Given the essential role of the cell envelope in mediating host‐pathogen interactions, the precise regulation of envelope‐associated genes is critical for bacterial adaptation within the host environment. One of the primary mechanisms by which bacteria regulate gene expression in response to environmental cues is through two‐component systems (TCS). These proteins enable bacteria to sense and respond to external changes, often modulating the expression of genes involved in cell envelope structure and function. A typical TCS consists of a sensor histidine kinase (HK) and its corresponding response regulator (RR) (West and Stock 2001). Upon detecting a chemical or physical signal, the HK undergoes autophosphorylation on a conserved histidine residue, transferring the phosphoryl group to an aspartate residue on the RR. This phosphoryltransfer event induces a conformational change in the RR, activating it to regulate the transcription of target genes (Gao et al. 2019). Through this signaling mechanism, TCSs are known to govern critical cellular processes including metabolism, motility, and stress adaptation, all of which support bacterial fitness and host interaction.

Although classical histidine kinases primarily function through direct phosphorylation of DNA‐binding response regulators, some HKs exhibit more complex regulatory mechanisms (Francis and Porter 2019; Willett and Crosson 2017). Among these, the HWE/HisKA2‐family kinases are particularly interesting due to their distinctive structural features (Dikiy et al. 2019, 2023; Herrou et al. 2017; Karniol and Vierstra 2004; Rivera‐Cancel et al. 2014; Swingle et al. 2025). These proteins, also known as HPK11 subfamily kinases (Grebe and Stock 1999), are hereafter referred to as HWE kinases. Several amino acid sequence motifs are unique to the HWE kinase family, and defining motifs present in most other HK families are missing in the HWE kinases (Herrou et al. 2017). Unlike standard histidine kinases that typically activate a single DNA‐binding response regulator, HWE kinases often engage in signaling processes involving multiple regulators, including other HWE kinases (Fiebig et al. 2019; Foreman et al. 2012; Kaczmarczyk et al. 2014, 2015; Kim et al. 2014; Lori et al. 2018; Reyes Ruiz et al. 2019; Wulser et al. 2022). This complex regulatory topology suggests that these proteins may function as sensory hubs, integrating multiple environmental signals to coordinate appropriate cellular responses.

A recent analysis focused on the identification of TCS genes that impact *B. ovis* cell envelope functions identified the HWE kinase, BOV_1602, as a genetic factor that strongly enhances resistance to sodium dodecyl sulfate (SDS), an anionic detergent (Chen et al. 2023). Orthologs of BOV_1602 in *B. abortus* (Kim et al. 2014) and *B. melitensis* (where the gene was named *phyK* (Gourley et al. 2015)) were previously assessed as regulators of the general stress response (GSR) gene regulatory system encoded by the *phyR*‐*nepR*‐*ecfG* locus. Both studies (Gourley et al. 2015; Kim et al. 2014) concluded that PhyK does not regulate *Brucella* GSR signaling under the tested conditions.

Given the crucial role of cell envelope integrity in *Brucella* stress adaptation and infection (Alakavuklar et al. 2023), further investigation of PhyK is necessary to clarify its involvement in detergent resistance, envelope physiology, and host interaction. To this end, we employed a combination of genetic, transcriptomic, immunological, and biochemical approaches to define the function and structure of *B. ovis* PhyK. Our study demonstrates that deletion of *phyK* enhances resistance to anionic detergents, increases susceptibility to cationic detergents, broadly reprograms transcription (especially genes linked to cell envelope functions) alters cell morphology, and modulates interactions with the host innate immune system. Furthermore, chromatographic analysis reveals that PhyK can form multiple stable oligomeric structures in solution, a property common to HWE kinases that may be integral to its signaling activity in the cell. These results expand our understanding of HWE‐family histidine kinases in bacterial stress adaptation and establish PhyK as a key regulator of *Brucella* envelope physiology and host interaction.

## 2 Results

### 2.1 Deletion of the HWE Histidine Kinase Gene, *phyK* , Broadly Impacts *B. ovis* Detergent Resistance

**FIGURE 1:**
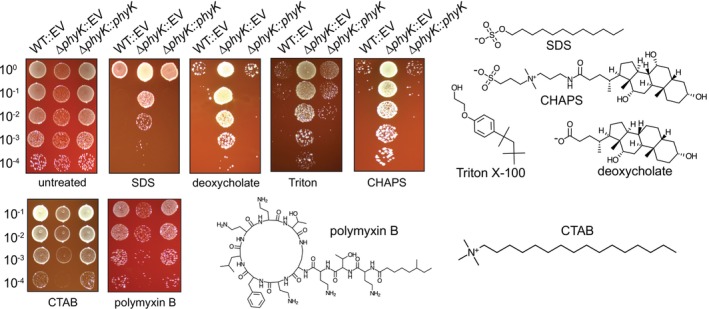
*phyK* deletion impacts *B. ovis* detergent resistance. The following strains were serially diluted and spotted onto TSA blood agar (TSAB): Wild type with an integrated empty vector (WT::EV), ∆*phyK* with an integrated empty vector (∆*phyK*::EV), and ∆*phyK* complemented with an integrated copy of *phyK* (∆*phyK*::*phyK*). Dilution factor is indicated on the left. TSAB plates contained SDS, sodium deoxycholate, Triton X‐100, CHAPS, CTAB, or polymyxin B. Dilution plating experiments were repeated three times with the same results, and one representative experiment is shown. Chemical structures of each detergent are shown.

*Brucella ovis* gene locus BOV_1602/BOV_RS07885 (hereafter referred to as *phyK*) encodes a predicted soluble, cytoplasmic histidine kinase. *phyK* is directly flanked by two small hypothetical genes at its 5′ end and the manganese uptake regulator *mur* (Anderson et al. 2009; Menscher et al. 2012) at its 3′ end; it is not directly adjacent to a response regulator gene on the *B. ovis* chromosome and is therefore considered an “orphan” histidine kinase. A recent study of TCS system genes that contribute to *B. ovis* fitness during in vitro envelope stress showed that deletion of *phyK* resulted in a significant increase in resistance to the anionic detergent, sodium dodecyl sulfate (SDS) (Chen et al. 2023). We therefore tested whether deletion of *phyK* influenced resistance to detergents with different chemical properties including the anionic bile acid deoxycholate, the non‐ionic detergent Triton X‐100, the cationic detergent cetyltrimethylamonium bromide (CTAB), and the zwitterionic detergent 3‐((3‐cholamidopropyl) dimethylammonio)‐a‐propanesulfonate (CHAPS) (Figure 1). Relative to the wild‐type (WT) strain, the in‐frame deletion strain (Δ*phyK*) was highly resistant to deoxycholate, Triton, and CHAPS when grown on solid medium (Figure 1). Conversely, Δ*phyK* was more sensitive to CTAB than WT. CTAB was the only cationic detergent in the group, so we further tested whether the mutant was sensitive to polymyxin B, which is a cationic antimicrobial peptide. Like CTAB, Δ*phyK* was more sensitive to polymyxin B than WT. These resistance/sensitivity phenotypes were genetically complemented by expression of the *phyK* gene from an ectopic locus (Figure 1).

**FIGURE 2:**
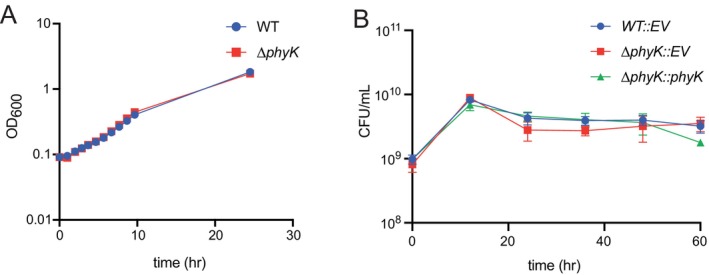
Deletion of *phyK* does not affect growth rate or cell survival in stationary phase (A) Growth curve of WT and ∆*phyK* in Brucella broth. (B) Recovered colony forming units (CFU) per mL of WT, ∆*phyK*, and ∆*phyK*::*phyK* cultures grown in Brucella broth. Experiments were repeated three times; error bars represent standard deviation of the three biological replicates.

To rule out the possibility that the detergent resistance phenotypes were influenced by differences in growth rate (Biselli et al. 2020; Kolter et al. 2022), we measured growth of WT and the Δ*phyK* mutant in broth. We observed no difference in growth rate or terminal culture density (Figure 2A). We further tested whether *phyK* deletion influenced *B. ovis* cell viability in stationary phase cultures by cultivating WT and *∆phyK* in broth and enumerating colony forming units (CFU) every 12 h for 60 h. CFUs recovered from ∆*phyK* mutant cultures were not significantly different from WT over this time course (Figure 2B). Thus, the increased resistance to anionic and neutral cell envelope disruptors as well as the increased sensitivity to cationic membrane disruptors we observed in a *B. ovis* ∆*phyK* mutant is not a consequence of differences in strain growth rate or fitness in stationary cultures. We conclude that the altered sensitivity of the ∆*phyK* mutant to diverse envelope‐disrupting agents reflects underlying changes in envelope structure or composition and is likely relevant to how *Brucella* spp. respond to host‐derived anionic compounds (e.g., short‐chain fatty acids) and cationic antimicrobial peptides encountered during infection and/or intracellular trafficking.

### 2.2 *phyK* Deletion Results in Large‐Scale Dysregulation of Membrane Transport and Bioenergy Systems

PhyK is a sensor histidine kinase and is therefore predicted to contribute to *B. ovis* detergent resistance through its effects on the regulation of transcription. To assess global transcript levels and define gene expression differences that may underlie the membrane stress resistance/sensitivity phenotypes of *ΔphyK*, we isolated RNA from WT and *ΔphyK* mutant strains and subjected these samples to RNA sequencing (RNA‐seq). The transcriptional profile of the Δ*phyK* strain was substantially different from wild type; over 700 genes were differentially expressed as a result of *phyK* deletion (|Fold change| > 2; false discovery rate (FDR) *p*‐value < 10^−6^; Table S1).

**FIGURE 3:**
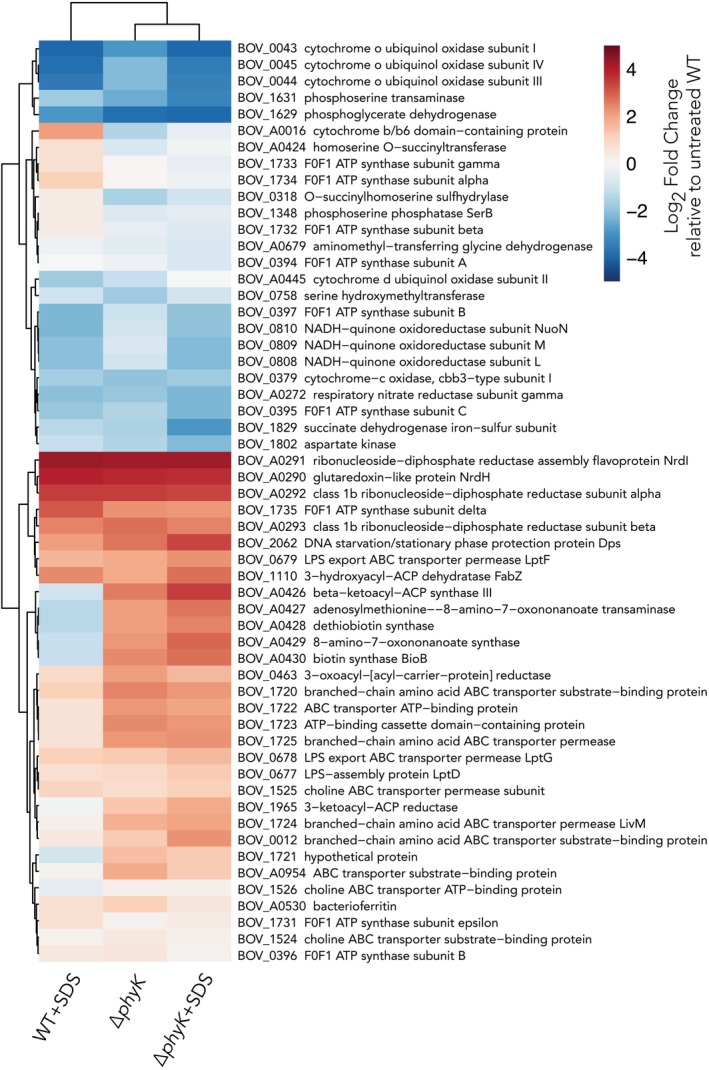
Heatmap of differentially expressed genes identified through gene set enrichment analysis (GSEA). A hierarchically clustered heatmap showing the log_2_ expression profiles of genes across three comparisons, each relative to untreated wild‐type (WT) *Brucella ovis* : (left column) WT treated with 0.0045% (w/v) SDS, (center column) untreated ∆*phyK*, and (right column) ∆*phyK* treated with 0.0045% SDS. The 56 genes shown belong to functional categories enriched by GSEA (FWER < 0.2; core enriched genes; see Table S1), including GO:0022904 (respiratory electron transport chain), GO:0009069 (serine family amino acid metabolic process), GO:0098533 (cell wall organization or biogenesis), GO:0006865 (amino acid transport), GO:0043190 (organic anion transport), and GO:0072523 (purine‐containing compound biosynthesis). Class 1b ribonucleotide reductase genes, though not falling within these categories, are included due to their strong differential expression in both SDS‐treated and ∆*phyK* backgrounds. The color scale (log_2_ fold change relative to untreated WT) is shown at the top right: Genes upregulated relative to untreated WT are represented in red, whereas downregulated genes are shown in blue.

GO term (Gene Ontology Consortium 2021) assignment followed by gene set enrichment analysis (GSEA) (Subramanian et al. 2005) revealed gene function classes that are significantly (FDR *p* < 0.05) dysregulated upon *phyK* deletion (Table S1). ATP‐binding cassette (ABC)‐dependent transport processes were strongly upregulated in Δ*phyK* relative to the WT. The primary substrate categories in this set of upregulated ABC transporters are predicted branched amino acid importers (*liv* system (Adams et al. 1990) homologs BOV_1720‐BOV_1725; BOV_0012), peptide import systems including glutathione (*gsiB*; BOV_A0954), lipopolysaccharide (LPS) export (*lptD*‐*lptG*‐*lptF*; BOV_0677‐BOV_0679), and choline transport (*choWV* homologs; BOV_1524‐BOV_1526) (Figure 3). The contributions of these upregulated transport systems to the observed detergent resistance phenotypes are not known, though choline import and LPS export have clear connections to phospholipid biosynthesis and outer membrane composition. The observed upregulation of fatty acid biosynthesis genes, including *fabG* (BOV_0463), *fabZ* (BOV_1110), and a 3‐ketoacyl‐ACP reductase (BOV_1965), along with the downregulation of predicted beta‐oxidation genes (BOV_A0744, BOV_A0746, BOV_A0392, BOV_1460) and the upregulation of the biotin biosynthesis operon (BOV_A0426‐BOV_A0430) in (Figure 3), indicate a shift toward net fatty acid biosynthesis in Δ*phyK*.

In contrast, a subset of genes involved in serine/glycine amino acid metabolism was significantly downregulated in Δ*phyK* (Table S1), as were multiple components of the *B. ovis* aerobic respiratory system. Downregulated genes included complex I (*nuo*), succinate dehydrogenase iron–sulfur subunit (BOV_1829), respiratory nitrate reductase subunit gamma (BOV_A0272), cytochrome o ubiquinol oxidase (BOV_0042–0045), cytochrome c bb3 oxidase (BOV_0375–0379), and cytochrome d ubiquinol oxidase (BOV_A0442‐0446) (Table S1). Transcription of the F1 sector subunits of ATP synthase remained largely unchanged, except for strong upregulation of the F1 delta subunit (BOV_1735). Transcription of the F0 subunits was downregulated in Δ*phyK*.

We also observed upregulation of the *ftrABC* iron importer (BOV_A0329‐BOV_A0331) and the iron homeostasis genes *bfr* (BOV_A0530) and *dps* (BOV_2062) in Δ*phyK*. Notably, the class 1b ribonucleoside‐diphosphate reductase operon (*nrdHIEF;* BOV_A0291‐BOV_A0293) was the most highly upregulated operon in Δ*phyK* compared to the WT strain (Figure 3). These genes catalyze the rate limiting step in generation of deoxyribonucleotides from ribonucleotides using a manganese cofactor. Previous studies have shown that *nrdHIEF* is highly upregulated in the naturally rough species *Brucella canis* during macrophage infection, but not in the smooth species *Brucella melitensis* under similar conditions (Eskra et al. 2012), raising the possibility that PhyK regulates a response to host infection that is specific to rough strains.

### 2.3 SDS Treatment and PhyK Deletion Induce Similar Transcriptomic Changes in *B. ovis*

Given that *phyK* deletion significantly altered *B. ovis* resistance to multiple detergents and antimicrobial peptides, including SDS, we next investigated whether SDS exposure triggered similar transcriptional changes. To test this, we conducted a parallel transcriptomic analysis of WT and Δ*phyK* cells grown in the presence of 0.00045% SDS. Growth of WT cells under these conditions resulted in widespread transcriptional reprogramming, with nearly one‐third of the genome exhibiting altered transcript levels compared to untreated cells (|Fold change| > 2; FDR *p*‐value < 10^−6^; Table S1). This large‐scale response shows that SDS induces substantial shifts in transcription.

**FIGURE 4:**
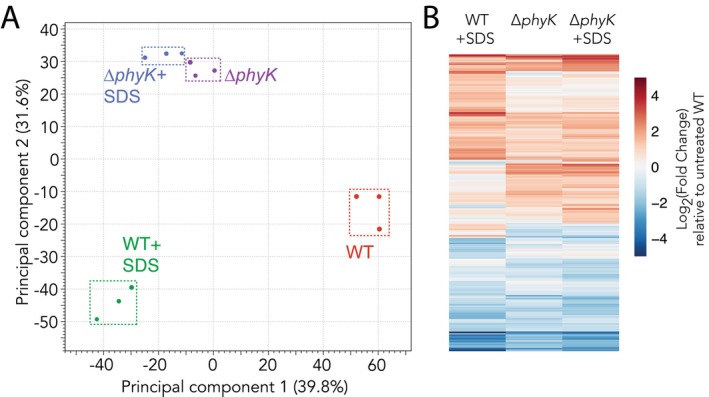
Genome‐scale transcriptional analysis of *B. ovis phyK* deletion and SDS treatment. (A) Principal component analysis (PCA) of the transcriptomic datasets of wild‐type (WT) or ∆*phyK* strains grown on a solid agar in the presence or absence of 0.0045% (w/v) sodium dodecyl sulfate (SDS) in the medium. The first two principal axes (PC1 and PC2) are shown and account for ~70% of the variance in the data. (B) Hierarchically clustered differential expression profiles of 1533 *B. ovis* genes (approximately 50% of genome) that are at least 2‐fold different and FDR *p* < 10^−6^ in any condition compared to untreated WT. Each row represents a gene. Color scale (log_2_ fold change relative to untreated wild type) is shown on far right. Genes upregulated relative to untreated WT are scaled in red; genes downregulated are scaled in blue.

To compare the global transcriptional responses to SDS treatment and *phyK* deletion, we first performed principal component analysis (PCA). The first two principal components (PC1 and PC2) together accounted for more than 70% of the total variance in our transcriptional datasets (Figure 4A). Along PC1, untreated WT samples separate from both SDS‐treated and ∆*phyK* mutant samples, suggesting an overlap in the transcriptional response to SDS treatment and *phyK* deletion. The ∆*phyK* samples, whether grown with or without SDS, cluster closely together, indicating that the transcriptional response to SDS is attenuated in ∆*phyK* cells compared to WT. The second principal component further separates WT and ∆*phyK* samples, highlighting transcriptional responses that are specific to *phyK* deletion.

We next clustered the differentially expressed genes that showed altered transcription in either untreated ∆*phyK* cells, SDS‐treated WT cells, or SDS‐treated ∆*phyK* cells compared to untreated WT cells (|Fold change| > 2; FDR *p*‐value < 10^−6^; Figure 4B). This gene set comprised nearly half the genome (1533 of 3171 genes). Consistent with the PCA analysis, we found that the global gene expression profile of SDS‐treated WT cells closely resembled that of the ∆*phyK* mutant, and that the transcriptional profile of ∆*phyK* cells remained largely unchanged in the presence or absence of SDS. Viewing the transcriptome at this scale suggests that *phyK* deletion triggers transcriptional reprogramming that mirrors the regulatory changes observed in SDS‐exposed cells, suggesting that ∆*phyK* mutants are transcriptionally ‘pre‐adapted’ to tolerate SDS.

Differential gene expression in response to SDS treatment primarily affected pathways involved in respiratory energy production, protein synthesis, and cell envelope remodeling. These patterns closely resembled those observed in the ∆*phyK* mutant but with some distinct functional differences. Like *phyK* deletion, SDS exposure led to widespread downregulation of genes encoding components of the electron transport chain, including multiple *nuo* genes (NADH‐quinone oxidoreductase subunits), cytochrome *c* oxidase, and cytochrome *o* ubiquinol oxidase. SDS also suppressed transcription of *atpB* and *atpC*, encoding F_0_F_1_ ATP synthase subunits, suggesting reduced energy production under detergent stress (Figure 3; Table S1). Additionally, SDS‐treated cells exhibited decreased expression of metabolic genes involved in glycolysis and pyrimidine biosynthesis, including *pgi* (glucose‐6‐phosphate isomerase), *pgk* (phosphoglycerate kinase), and *pyrC* (dihydroorotase), further supporting a shift away from energy‐intensive biosynthetic processes (Table S1).

In contrast, SDS treatment strongly upregulated genes involved in translation and cell envelope homeostasis. A subset of ribosomal protein genes (e.g., 30S subunits *rpsG, rpsL, rpsK*; 50S subunits *rplY, rplE, rplM, rplJ, rplL*) were significantly induced, suggesting an altered translation profile in response to detergent stress. Upregulation of tRNA ligases, including those for serine, valine, and methionine, further supports a shift in protein synthesis. SDS exposure also activated genes involved in ABC transport and LPS biosynthesis, indicating restructuring of the cell envelope transport system (Table S1).

Together, these results indicate that SDS exposure triggers a global regulatory response that reduces energy metabolism and primary biosynthetic pathways while impacting protein synthesis and cell envelope remodeling. The similarity between the transcriptional changes induced by SDS and those observed in the ∆*phyK* mutant suggests that *phyK* deletion elicits a preemptive gene expression state that buffers against SDS‐induced membrane stress. This transcriptional “pre‐adaptation” may contribute to the increased resistance of ∆*phyK* cells to anionic detergents like SDS.

### 2.4 Deletion of *phyK* Alters the Morphology of *B. ovis* and the Host Inflammatory Response

**FIGURE 5:**
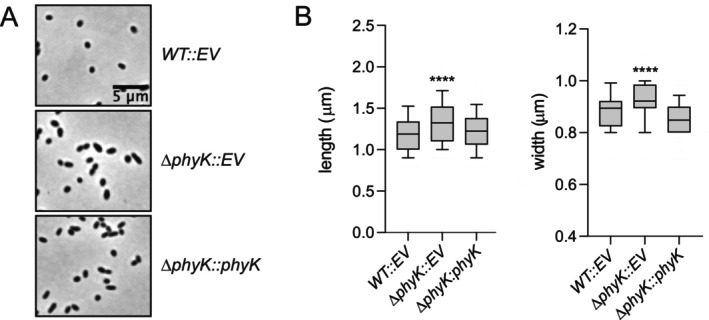
Deletion of *phyK* results in longer and wider *B. ovis* cells. (A) Representative phase contrast micrographs (630× magnification) of WT and *∆phyK* strains harboring either an integrated empty vector (::EV) or an integrated plasmid bearing *phyK* under its native promoter (::*phyK*). (B) Length and width analysis of WT::EV (*n* = 267 cells), ∆*phyK*::EV (*n* = 425 cells), and the complemented *∆phyK*::*phyK* strain (*n* = 522 cells). Mean is shown as a horizontal line in the box (25th‐75th percentile); whiskers capture from 10th‐90th percentile. Statistical significance was calculated using one‐way ANOVA, followed by Dunnett's multiple comparison test to WT::EV (*p* < 0.0001, ****).

Our results indicate that PhyK is a key regulator of *Brucella* cell envelope function, as evidenced by its role in detergent resistance (Figure 1) and its broad influence on the transcription of genes associated with membrane integrity and stress responses (Figures 3 and 4; Table S1). Given the extensive transcriptional changes observed in the Δ*phyK* strain, we hypothesized that this mutant would exhibit morphological differences compared to WT. Indeed, quantitative measurements of phase‐contrast micrographs confirmed that Δ*phyK* cells are significantly longer and wider than WT cells (Figure 5).

This morphological defect is reminiscent of cell width changes observed in *E. coli* strains with hyperactive Cpx envelope stress signaling (Delhaye et al. 2016; Pogliano et al. 1998), which are linked to Cpx‐mediated regulation of peptidoglycan metabolism (Delhaye et al. 2016). Although PhyK does not appear to be a major regulator of peptidoglycan metabolism, it significantly upregulates *phiA*, a peptidoglycan hydrolase inhibitor (Del Giudice et al. 2019) (Table S1). Additionally, transcriptomic data provide evidence that the Δ*phyK* strain has disrupted fatty acid biosynthesis, a process linked to cell size control in *E. coli* (Yao et al. 2012). Further studies are needed to determine the specific mechanisms by which *phyK* deletion alters cell morphology.

**FIGURE 6:**
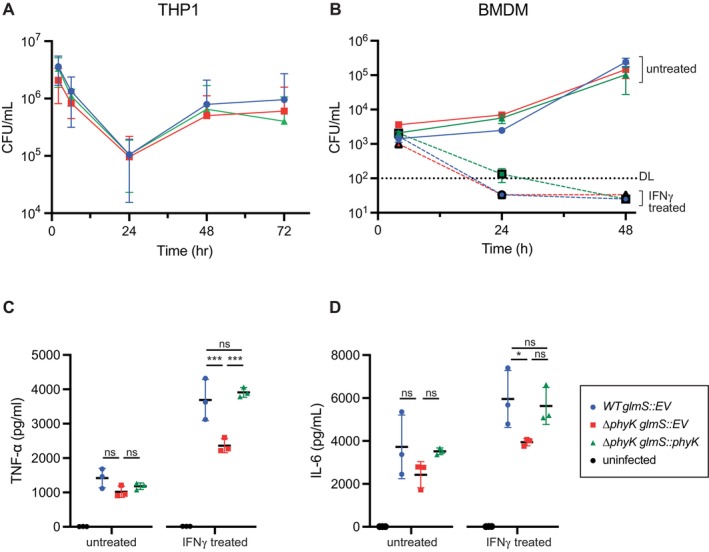
Deletion of *phyK* does not impair intracellular survival but reduces immune stimulation during infection. Colony forming units (CFU) of *B. ovis* strains recovered from (A) human THP‐1 macrophage‐like cells at 2, 6, 24, 48, and 72 h post‐infection or (B) bone marrow‐derived macrophages (BMDM) either untreated (i.e., resting) or activated with interferon‐γ (IFNγ) at 4, 24, or 48 h post‐infection. The assay detection limit (DL) is 100 CFU/mL. (C) TNF‐α and (D) IL‐6 cytokines released into the supernatant by resting and activated BMDMs infected with the indicated *B. ovis* strains for 24 h. Values in C and in D were compared using a 2‐way ANOVA followed by Tukey's post‐test to test for differences between treatment and strains. *p* Values for the comparisons between strains are shown; *p* > 0.05 n.s., *p* < 0.05 *, *p* < 0.01 **, *p* < 0.001 ***. In all panels, WT *B. ovis* bearing an empty vector (EV) integrated at the *glmS* locus is depicted by blue circles, ∆*phyK* bearing an integrated empty vector are red squares, and ∆*phyK* bearing a complementing copy of *phyK* integrated at the *glmS* locus are green triangles, as in key. Data represent at least two independent experiments, and error bars indicate the standard deviation of replicates.

Considering the pronounced differences in detergent resistance and gene expression between Δ*phyK* and WT, including the dysregulation of genes within the Type IV Secretion System (T4SS) (Table S1), we hypothesized that the Δ*phyK* strain would exhibit a fitness defect in a macrophage infection model. Furthermore, transposon insertions in the *B. abortus phyK* ortholog (BAB_1669) were previously shown to attenuate survival in RAW 264.7 macrophages (Sternon et al. 2018). However, deletion of *phyK* in *B. ovis* did not result in a significant defect or advantage in either macrophage entry (2 h post‐infection) or intracellular replication/survival (6, 24, 48, and 72 h post‐infection) in THP‐1 macrophage‐like cells compared to WT (Figure 6A).

We further enumerated bacterial CFU at 4, 24, and 48 h post‐infection in murine primary bone marrow‐derived macrophages (BMDMs), using both resting and interferon‐γ–activated cells. Again, there was no significant difference between wild‐type and Δ*phyK* CFU in resting BMDMs; though, as expected, overall bacterial burden was lower in this primary cell line. Interferon‐γ treatment of BMDMs resulted in almost complete killing of all strains by 24 h (Figure 6B). Given the pronounced differences between the cell envelopes of the Δ*phyK* mutant and the wild‐type strain, we tested whether the mutant had altered immune stimulatory properties in resting and activated BMDMs. To assess this, we measured levels of the pro‐inflammatory cytokines TNF‐α and IL‐6 at 24 h post‐infection. Cytokine levels were significantly lower in activated BMDMs infected with the Δ*phyK* mutant compared to those infected with wild‐type or the genetically complemented Δ*phyK* strain (Figure 6C,D). These results indicate that although *phyK* deletion does not impair intracellular fitness in the macrophage models tested, it diminishes the innate immune response against *B. ovis* . It is possible that this altered interaction with the immune system enhances the ability of the Δ*phyK* strain to persist within the intracellular niche, offsetting the fitness cost of disrupted envelope homeostasis caused by *phyK* deletion.

### 2.5 PhyK Adopts Multiple Stable Oligomeric States in Solution

We have shown that PhyK plays a key role in the regulation of *B. ovis* cell envelope physiology, broadly impacting the expression of genes that influence resistance to membrane‐disrupting agents and that impact host immune response. Although most histidine kinases function as obligate homodimers (Ashenberg et al. 2011; Hidaka et al. 1997), prior studies have shown that members of the HWE kinase family can adopt diverse oligomeric states, including monomers (Dikiy et al. 2019; Rivera‐Cancel et al. 2014) and homohexamers (Wojnowska et al. 2013). Given this structural variability and the potential for complex regulatory mechanisms among HWE kinases to regulate the *Brucella* cell envelope, we sought to determine the oligomeric state(s) of PhyK in solution.

**FIGURE 7:**
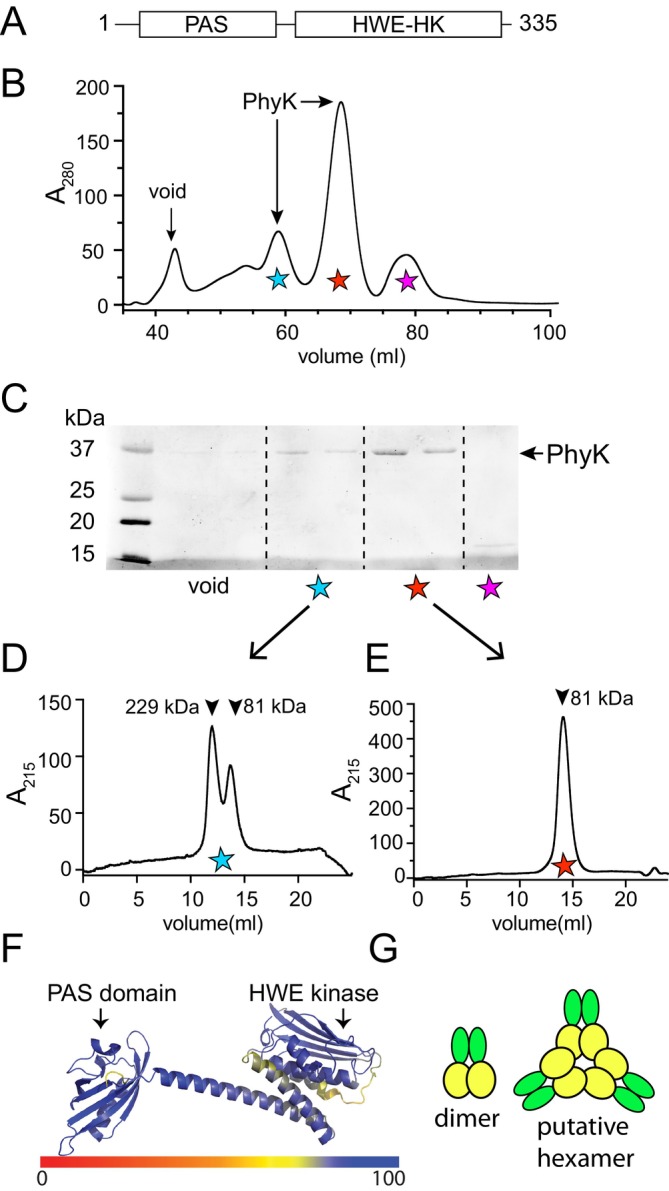
Evidence for multiple oligomeric forms of PhyK in solution. (A) Schematic of the PhyK protein domain structure with an N‐terminal PAS domain and a C‐terminal HWE‐family kinase domain (HWE‐HK); PhyK has no predicted transmembrane domains. (B) Size‐exclusion chromatography (SEC) profile of purified *B. ovis* PhyK protein resolved on a Superdex 200 16/600 column. Multiple elution peaks suggested the presence of multiple oligomeric states. (C) Coomassie‐stained SDS‐PAGE gel of fractions from each of the starred peaks in the SEC profile in B. Uncropped gel image presented in Figure S1. (D) The estimated ~250 kDa peak fraction (blue star) eluted as two peaks after resolution on a Superdex 200 10/300 column, consistent with putative PhyK hexamers and dimers. (E) SEC of the ~90 kDa peak on a Superdex 200 10/300 column resolved a single peak, consistent with the apparent size of a PhyK dimer. (F) Predicted tertiary structure of a PhyK monomer. Prediction confidence score is shown on a scale from red (low) to blue (high). (G) PAS‐HWE kinases may adopt a range of oligomeric states, including dimers and higher‐order assemblies. The structural basis of higher‐order interactions are not known for PhyK, but PAS domains have been modeled green and HWE kinase domains yellow for postulated dimer and hexamer forms.

PhyK is a 335‐residue protein composed of an N‐terminal PAS domain (Henry and Crosson 2011; Taylor and Zhulin 1999) and a C‐terminal HWE kinase domain (Figure 7A), with no predicted transmembrane domains (Gumerov et al. 2024). Recent structural analyses of PAS‐HWE kinases revealed significant variability in oligomerization, with some existing as monomers and others forming stable homodimers (Dikiy et al. 2023). Notably, certain members of this group were shown to exist as mixtures of monomers and dimers in solution, suggesting that their quaternary structure may be dynamic. To investigate the oligomeric state(s) of PhyK, we heterologously expressed and purified the protein, then analyzed its solution behavior using size‐exclusion chromatography (SEC).

When resolved on a preparative Superdex 200 16/600 column at 60 μM, PhyK eluted in three distinct peaks, corresponding to apparent molecular weights of > 600 kDa (void volume), ~250 kDa, and ~ 90 kDa (Figure 7B). SDS‐PAGE analysis confirmed that these peaks represent different homo‐oligomeric forms of full‐length PhyK, rather than degradation products or contaminants (Figure 7C and Figure S1). To further refine these molecular weight estimates, we collected fractions containing the ~250 and ~ 90 kDa peaks and subjected them to high‐resolution SEC using a Superdex 200 10/300 column. At lower protein concentrations (7–14 μM), the ~250 kDa fraction separated into two distinct peaks, consistent with putative PhyK hexamers and dimers (Figure 7D), while the ~90 kDa fraction resolved as a single peak corresponding to the expected size of a dimer (Figure 7E). These results provide evidence that PhyK primarily exists as a dimer but can also form stable higher‐order oligomers at micromolar concentrations (Figure 7F–G).

## 3 Discussion

We have identified the orphan HWE‐family kinase PhyK as an important regulator of the *B. ovis* cell envelope. Loss of *phyK* triggers widespread transcriptional remodeling in transport, metabolism, and respiration pathways, confers resistance to anionic detergents, increases sensitivity to cationic peptides, perturbs cell morphology, and dampens the innate immune response to *B. ovis* . This study thus illuminates roles for PhyK in envelope stress responses and host interaction.

### 3.1 Parallels With the Cpx System

The transcriptional impact of *phyK* deletion is aligned with transcriptional changes induced by SDS treatment of wild‐type *B. ovis* cells (Figures 3 and 4; Table S1). This suggests that the *phyK* mutant is transcriptionally pre‐adapted to withstand anionic detergent stress. There are striking parallels between gene expression changes observed in the *phyK* mutant and those observed in *Escherichia coli* mutants that activate the Cpx envelope stress response system (Raivio 2014) including reduced transcript levels of a succinate dehydrogenase subunit, NADH dehydrogenase, and the cytochrome *bo* oxidase (Raivio et al. 2013). One of the early reported phenotypes of Cpx activation in *E. coli* was a defect in branched‐chain amino acid biosynthesis (McEwen and Silverman 1980), which resulted from a defect in acetohydroxyacid synthase function (Sutton et al. 1982). In *B. ovis* , *phyK* deletion leads to strong upregulation of predicted branched‐chain amino acid import systems, along with increased expression of acetolactate synthase (*BOV_1346*) (Figure 4 and Table S1), suggesting *phyK* and *cpx* regulate the expression of related metabolic systems in response to envelope stress. It is possible that import and synthesis of branched‐chain amino acids in *Brucella* supports the biosynthesis of branched‐chain fatty acids, which modulate membrane properties (Mostofian et al. 2019) and have been linked to stress tolerance (Giotis et al. 2007). The envelope remodeling evident in the Δ*phyK* mutant may alter the surface presentation of immune‐stimulatory molecules such as outer membrane proteins or lipoproteins, contributing to the differential inflammatory cytokine responses of activated macrophages to the wild‐type and Δ*phyK* strains (Figure 6).

### 3.2 A Role for *phyK* in GSR Regulation by PhyR and σ ^EcfG^ ?

Previous studies in *Brucella* spp. found no evidence that *phyK* directly regulates transcription through the general stress response (GSR) sigma factor σ^EcfG^ (RpoE1) (Gourley et al. 2015; Kim et al. 2014). However, in *Sinorhizobium meliloti* , a related HWE kinase RsiC was shown to both activate and repress GSR transcription (Sauviac and Bruand 2014), suggesting that PhyK could play a more complex regulatory role in the GSR than previously recognized. Our transcriptomic data present a somewhat conflicting picture regarding the influence of PhyK on *B. ovis* σ^EcfG^ activity.

Transcriptomic analyses of a *B. abortus ecfG* deletion mutant identified *dps*, *ba14K*, and *rpoH* as the three most highly activated σ^EcfG^ targets, with their expression decreasing approximately 100‐fold upon *ecfG* deletion (Kim et al. 2014). In contrast, our RNA‐seq analysis of the *B. ovis* Δ*phyK* mutant revealed opposing expression profiles at these loci. Specifically, *dps* (*BOV_2062*) transcript levels increased approximately sixfold in the absence of *phyK*, suggesting that PhyK negatively regulates *dps* expression. Conversely, *ba14K* (*BOV_A0688*) and *rpoH* (*BOV_1597*) exhibited modest decreases in expression, indicating that PhyK may have a limited activating role in their transcription. These results raise the possibility that PhyK influences σ^EcfG^ activity through phosphorylation and dephosphorylation of its anti‐anti‐σ factor PhyR, potentially acting as part of a broader network of *Brucella* HWE‐family kinases (Kim et al. 2013, 2014; Luebke et al. 2018) that coordinate stress responses through combinatorial signaling to PhyR. Future experiments will clarify how the soluble, cytoplasmic kinase PhyK exerts such broad transcriptional effects in *Brucella* and whether this regulation is mediated through the PhyR‐NepR‐σ^EcfG^ GSR partner switching system (Fiebig et al. 2015; Francez‐Charlot et al. 2015).

Our biochemical analysis provides evidence that PhyK can adopt multiple stable oligomeric states in solution, existing primarily as a dimer but also forming higher‐order oligomers at micromolar concentrations (Figure 7). As discussed above, HWE‐family kinases exhibit diverse quaternary structures (Dikiy et al. 2023), and this structural plasticity could have functional implications for PhyK‐dependent transcriptional regulation. One possibility is that the ability of PhyK to transition between oligomeric states facilitates interactions with other HWE kinases involved in *Brucella* cell envelope regulation. Such dynamic oligomerization has been postulated to promote cooperative or hierarchical signaling cascades that influence σ^EcfG^‐dependent transcription (Lori et al. 2018; Reyes Ruiz et al. 2019). This raises the possibility that PhyK functions as part of a broader system of GSR kinases, integrating multiple stress signals to coordinate transcriptional responses. Studies testing whether the oligomeric state of PhyK influences its regulatory activity and whether it physically interacts with other histidine kinases are ongoing.

### 3.3 Respiratory Shifts and Potential Links to Metal Homeostasis

The marked downregulation of iron‐requiring aerobic respiratory genes (*nuo, cyo, cyd*) and upregulation of the manganese‐requiring ribonucleotide reductase genes (*nrdHIEF*) hints at the possibility that SDS treatment and *phyK* deletion cue an iron limitation response. However, we did not observe increased expression of genes expected to be activated upon iron limitation in *Brucella* (Roop 2nd 2012), with the exception of the *ftr* transport system (Elhassanny et al. 2013). Transcription of the oxidative stress regulator, *oxyR* (BOV_A0321) (Kim and Mayfield 2000) was modestly upregulated in the SDS‐resistant *phyK* mutant suggesting that *phyK* deletion may impact production of peroxide in *B. ovis* . However, *oxyR* expression was unchanged in SDS‐treated wild‐type cells and expression of the catalase *katE* (BOV_A0322) (Kim and Mayfield 2000), an *oxyR*/H_2_O_2_‐induced enzyme, remained unchanged in the mutant and was downregulated in wild‐type cells treated with SDS. So, while it remains possible that the large‐scale changes in the expression of respiratory complexes upon *phyK* deletion or SDS treatment influence reactive oxygen species (ROS) in the cell, the transcriptomic data presented herein (Table S1) suggest that fluctuations in H_2_O_2_ levels are unlikely to be a major factor driving the observed gene expression patterns and cell viability phenotypes. Additional studies are needed to directly assess how *phyK* deletion influences cellular respiration, metal homeostasis, and reactive oxygen production.

## 4 Conclusion

Our results establish PhyK as an important regulator of cell envelope physiology in *B. ovis* that impacts environmental stress responses and host immune responses. Although its molecular signaling mechanism remains undefined, deletion of *phyK* leads to large‐scale changes in transcription that enhance resistance to anionic envelope disruptors but sensitize cells to cationic agents. Despite pronounced envelope stress survival phenotypes in vitro, the ∆*phyK* strain did not have a fitness defect in ex vivo macrophage infection models. However, ∆*phyK* elicits a muted pro‐inflammatory response in activated BMDMs relative to the wild‐type strain, revealing a link between PhyK‐dependent cell envelope regulation and host immune stimulation. Further defining how PhyK modulates transcription, whether through σ^EcfG^, other regulators, or post‐transcriptional mechanisms, will be key to understanding how *Brucella* maintains envelope homeostasis in response to stressors encountered during infection.

## 5 Experimental Procedures

### 5.1 Growth Conditions and Strains

All *Brucella ovis* ATCC25840 and *phyK* (BOV_1602) mutant derivative strains that were previously described in (Chen et al. 2023) were grown on Tryptic soy agar (BD Difco) plus 5% sheep blood (Quad Five) (TSAB) or in Brucella broth at 37°C with 5% CO_2_ supplementation. Liquid cultures were aerated by rolling. When needed, the growth medium was supplemented with 50 μg/mL kanamycin and/or detergents/membrane disruptors—compounds and concentrations noted below.

### 5.2 Agar Plate Detergent Stress Assays and Liquid Polymyxin B Stress Assay

After 2 days of growth on TSAB or TSAB with 50 μg/mL kanamycin, *B. ovis* cells were collected and resuspended in sterile phosphate‐buffered saline (PBS) to an OD_600_ = 0.3. Each strain was 10‐fold serially diluted in PBS. 5 μL of each dilution was spotted onto either TSAB, TSAB containing 0.0045% SDS, 0.008%–0.009% sodium deoxycholate, 0.0065%–0.008% Triton, or 0.1% CHAPS, and 3 μL onto TSAB containing 0.011% CTAB. Growth on plates was documented photographically after 3 days of incubation for plain TSAB or 4 days for TSAB + detergent.

For polymyxin B stress assays, *B. ovis* cells were collected and resuspended in 1 mL of Brucella broth at an OD_600_ = 0.3. Cells were split and one portion was treated with 1 mg/mL polymyxin B, and the other was untreated. Both treated and untreated groups were incubated at 37°C with 5% CO_2_ supplementation for 80 min. Each culture was then 10‐fold serially diluted in PBS and 5 μL of each dilution was spotted onto TSAB to enumerate CFU after treatment. After 3 days of incubation for untreated group and 4 days for treated group, growth was documented photographically.

### 5.3 Growth Curve

Strains were inoculated from freezer stocks onto TSAB plates and grown for 2 days. Cells were inoculated in 8 mL Brucella broth and incubated on rotor overnight. The next day, cells were back diluted to OD_600_ = 0.05 in 9 mL Brucella broth at time = 0 h. Optical density at 600 nm was measured every hour for the first 9 h and again at 24 h.

### 5.4 Long‐Term Stationary Phase Survival Assay

After 2 days of growth on TSAB containing kanamycin, *B. ovis* cells were collected and resuspended in 8 mL Brucella broth and incubated on a rotor overnight. The next day, cells were back diluted to OD_600_ = 0.05 in 9 mL Brucella broth at time = 0 h. At 12 h intervals, 100 μL of culture was serially diluted in PBS and 5 μL of each dilution was plated onto TSAB. Plates were incubated for 3 days and CFU were enumerated.

### 5.5 RNA Extraction and Sequencing

Wild‐type and *∆phyK B. ovis* strains were initially grown on TSAB for 2 days. Cells were then scraped from the TSAB plates, resuspended in Brucella broth and adjusted to OD_600_ = 0.3. 200 μL of each diluted culture was plated onto either TSAB or TSAB +0.004% SDS and spread evenly with sterile cotton swabs. WT and *∆phyK* grown on TSAB and *∆phyK* grown on TSAB +0.004% SDS were incubated for 3 days. WT grown on TSAB +0.004% SDS was incubated for 4 days. Cells were collected from plates by scraping and resuspended in 1 mL TRIzol and stored at −80°C until RNA extraction. The extraction process was the same as described in (Chen et al. 2023). Briefly, samples were thawed and incubated at 65°C for 10 min. 200 μL of chloroform was added to each sample and mixed by vortexing for 15 s. Samples were then incubated at room temperature for 5 min, and liquid phases were separated by centrifugation at 17,000 *g* for 15 min at 4°C. The aqueous layer was transferred into a fresh tube, mixed with 500 mL of isopropanol and stored at −20°C for 2 h. After thawing, samples were centrifuged at 13,000 *g* for 30 min at 4°C to pellet the RNA. Supernatants were discarded and the pellets were washed with 1 mL of ice cold 70% ethanol. Samples were centrifuged at 13,000 *g* at 4°C for 5 min. Supernatants were discarded and pellets were air dried. Pellets were resuspended in 100 μL RNAse‐free H_2_O and incubated at 60°C for 10 min. RNA samples were DNase treated and RNA isolated with the RNeasy Mini kit (Qiagen). RNA samples were sequenced at Microbial Genome Sequencing Center (Pittsburgh, PA) on an Illumina NextSeq 2000 (Illumina). Libraries for RNA‐seq were prepared using Illumina Stranded RNA library preparation with RiboZero Plus rRNA depletion.

### 5.6 RNA‐Seq Analysis

All RNA‐seq analysis was conducted in CLC Genomics Workbench 21.0.4 (Qiagen) using the RNA‐seq default settings. Reads were mapped to the reference genome ( *Brucella ovis* ATCC 25840, GenBank accessions NC_009504 and NC_009505). Differential expression for RNA‐seq workflow was used to calculate the fold change and statistical significance of all annotated genes in mutant and SDS treatment conditions versus WT untreated. GO terms (Gene Ontology Consortium 2021) were assigned to each gene in the *B. ovis* ATCC 25840 genome using BioBam Cloud BLAST (Table S1). We then implemented gene set enrichment analysis (GSEA) (Subramanian et al. 2005), to identify gene function classes that are significantly enriched in up‐ or downregulated genes upon *phyK* deletion or SDS treatment. Raw and processed RNA‐seq data are publicly available in the NCBI GEO database at accession GSE262183.

### 5.7 Principal Component Analysis of RNA‐Seq Data

RNA‐seq count data were imported from a CSV file. Normalization was performed using the counts‐per‐million (CPM) method to account for differences in sequencing depth across samples. The data were then log transformed (log_2_(CPM + 1)) to stabilize variance and the log‐transformed data were standardized to have a mean of zero and unit variance using the StandardScaler function. Principal Component Analysis (PCA) was performed to reduce dimensionality and identify major sources of variation in the dataset. The analysis retained three principal components, capturing the most variance in the data. The PCA results were visualized as scatterplots for the first two principal components (PC1, PC2), which accounted for ~70% of the variance. Analysis was conducted using Python, leveraging the pandas, numpy, scikit‐learn, and seaborn libraries for data manipulation, PCA computation, and visualization.

### 5.8 Macrophage Infection Assay and Cytokine Measurements

THP‐1 cells were grown in Roswell Park Memorial Institute Medium (RPMI) + 10% heat inactivated fetal bovine serum (FBS) at 37°C with 5% CO_2_ supplementation. Three days prior to infection, 10^5^ THP‐1 cells were seeded in a 96‐well plate at a concentration of 10^6^ cells/mL in 100 μL/well of fresh RPMI +10% FBS with an addition of 50 ng/mL phorbol 12‐myristate‐13‐acetate (PMA) to induce differentiation.

For bone marrow‐derived macrophages (BMDMs), femurs from C57BL6/J (Jackson Lab #000664) were isolated, and bone marrow was harvested following centrifugation of bones cut on one side. Bone marrow was then cultured in complete RPMI 1640 media containing 30 ng/mL M‐CSF (R&D Systems) for 7 days in untreated 15 cm dishes. Cells were then split for experiments and treated with or without 25 ng/mL murine IFN‐gamma (R&D Systems). Experimental protocols were approved by the Institutional Animal Care and Use Committees at Michigan State University (animal use form [AUF] no. PROTO202500031).

After 3 days at 37°C in 5% CO_2_, *B. ovis* cells were resuspended at a concentration of 10^8^ CFU/mL (OD_600_ = 0.15) in RPMI and 100 μL were added to macrophages to give a multiplicity of infection (MOI) of 100. The plate containing *B. ovis* and macrophages was centrifuged at 150 *g* at room temperature for 5 min and incubated at 37°C with 5% CO_2_ for 1 h. Media was removed and fresh media containing 50 μg/mL gentamicin was added to kill extracellular *B. ovis* that were not internalized. The plate was incubated for another hour at 37°C with 5% CO_2_. Media was removed and fresh media containing 25 μg/mL gentamicin was added to each well except for wells to be immediately harvested 2 h post‐infection. At specified time points post‐infection, *B. ovis* were enumerated by removing the media and washing the cells with PBS once and incubating at 37°C for 10 min. Then PBS was removed and 200 μL dH_2_O was added to each well and the plate was incubated at room temperature for 10 min to lyse the macrophages. *B. ovis* cells were collected and 10‐fold serial diluted in PBS and spotted on TSAB. Plates were incubated for 3 days and CFU were enumerated.

Cytokine concentrations in culture supernatants were quantified using commercial ELISA kits (R&D Systems DY410 and DY406).

### 5.9 Phase Contrast Microscopy

Wild‐type and mutant *B. ovis* cells were grown on TSAB supplemented with kanamycin and incubated for 2 days. Cells were resuspended in milliQ H_2_O and 1 μL of cells were spotted on an agarose pad (1% agarose in PBS) on a cover slide and imaged on a Leica DMI 6000 microscope using phase contrast with an HC PL APO 63×/1.4 numeric aperture (NA) oil Ph3 CS2 objective. Images were captured with an Orca‐ER digital camera (Hamamatsu) controlled by Leica Application Suite X (Leica). Cell areas were measured from phase contrast images using BacStalk (Hartmann et al. 2020).

### 5.10 PhyK Protein Expression, Purification, and Assessment of Oligomeric State

Full‐length *B. ovis phyK* was amplified with KOD Xtreme DNA polymerase and fused into a pET23b‐His_6_‐SUMO expression vector using FastCloning. Briefy, *phyK* was amplified with the following primers, 5′gaacagaccggtggaATGACTGCGCCGAAGCCA and 5′agcagccggatctcaTGAATCGAATTGTTCGGTCGGG, where the lower case letters overlap with the plasmid sequence. Linearized pET23b‐His6‐SUMO was amplified from miniprepped plasmid with the following primers, 5′tgagatccggctgctaacaaag and 5′tccaccggtctgttctctgt, before digesting the template with DpnI. Then, the amplified *phyK*‐containing fragment and the amplified linearized plasmid with overlapping ends were co‐transformed into competent *E. coli* TOP10 (Invitrogen). Plasmid bearing colonies were selected on LB agar containing 100 μg/mL carbenicillin. The sequence of the vector was confirmed by whole plasmid sequencing before it was transformed into chemically competent *E. coli* BL21 (DE3). The resulting strain was grown in 1 L of LB medium at 37°C. When the optical density reached OD_600_ = 0.5, expression was induced with 0.5 mM isopropyl b‐D‐1‐thiogalactopyranoside. The cultures were shifted to 16°C and grown overnight. Cells were then harvested by centrifugation and cell pellets were stored at −80°C. For protein purification, cell pellets were resuspended in 25 mL lysis buffer (25 mM Tris pH 8, 125 mM NaCl 10 mM imidazole) spiked with 1 mM phenylmethylsulphonyl fluoride (PMSF) and 5 μg/mL DNase I. Cells were lysed through sonication on ice until the lysates were clear. Lysates were centrifuged at 27,000 RCF for 30 min to remove cell debris. The supernatant was applied to a gravity drip column containing 3 mL of Ni‐nitrilotriacetic acid (NTA) superflow resin (Qiagen) that had been pre‐equilibrated with lysis buffer. The resin was washed with 20 mL lysis buffer, followed by 50 mL wash buffer (25 mM Tris pH 8, 125 mM NaCl 30 mM imidazole), and the proteins were collected in 10 mL of elution buffer (25 mM Tris pH 8, 125 NaCl, 300 mM imidazole). The elusion was dialyzed against buffer without imidazole, and the His_6_‐SUMO tag was cleaved with ubiquitin‐like‐specific protease 1 (Ulp1) overnight at 4°C in dialysis buffer (25 mM Tris pH 8, 125 mM NaCl). The next day, the digested protein was mixed with NTA superflow resin and applied to a gravity drip column. The cleaved protein was collected in the flow through and concentrated to a volume of 900 μL using an Amicon Ultra protein concentrator with a 10 kDa molecular weight cutoff. The sample was stored at 4°C until use.

Protein concentration was determined by UV absorption spectroscopy using a calculated extinction coefficient of 46,410 M^−1^ cm^−1^. 900 μL of 62 μM PhyK sample was resolved on a Superdex 200 16/60 HiPrep Column, which yielded two major fractions that contained PhyK. 450 μL of 40 μM PhyK from the largest of two peaks was subsequently resolved on a Superdex 200 10/30 analytical gel filtration column. 450 μL of 15 μM PhyK from the smaller of these peaks was subsequently resolved on a Superdex 200 10/30 analytical gel filtration column.

## Author Contributions

**Xingru Chen:** conceptualization, investigation, writing – review and editing, validation, visualization, formal analysis, writing – original draft. **Emily Perez:** formal analysis, investigation, writing – review and editing. **Eleanor C. Scheeres:** investigation, formal analysis. **Rosemary Northcote:** investigation, writing – review and editing. **Aretha Fiebig:** conceptualization, formal analysis, visualization, writing – review and editing, supervision, methodology. **Andrew J. Olive:** conceptualization, writing – review and editing, supervision, funding acquisition, formal analysis, resources, methodology. **Sean Crosson:** conceptualization, formal analysis, visualization, writing – original draft, writing – review and editing, supervision, methodology, funding acquisition, resources.

## Supporting information


Data S1

Table S1

## Data Availability

The data that support the findings of this study are openly available in GEO at https://www.ncbi.nlm.nih.gov/geo/, reference number GSE262183.
