# Supplementary material for: An HWE‐Family Histidine Kinase Modulates Brucella Cell Envelope Properties and Host Innate Immune Response
Source: Mol Microbiol. 2025 Jun 26;124(3):245–58. doi: 10.1111/mmi.70006 (PMC12354254; doi:10.1111/mmi.70006)
Supplement: Supplementary file 1 — Data S1 [file MMI-124-245-s001.docx]

Supplemental Figures


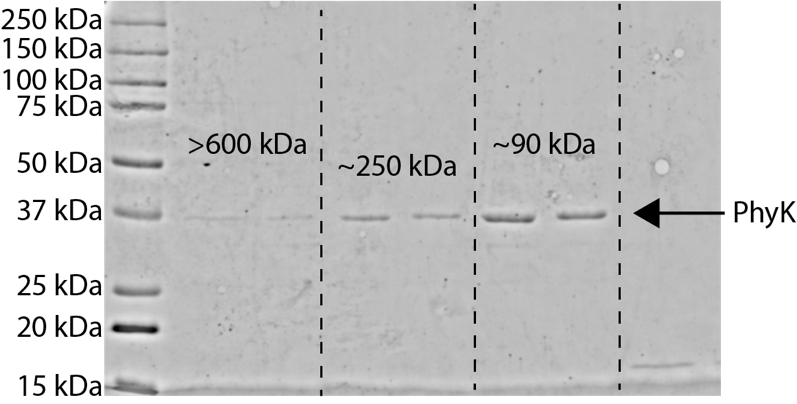


Figure S1. Full, coomaasie-stained SDS-PAGE gel of *Brucella ovis* PhyK fractions eluted from Superdex 200 HiPrep 16/600 (cropped gel presented in Figure 7).
